# Comparison of Fish, Krill and Flaxseed as Omega-3 Sources to Increase the Omega-3 Index in Dogs

**DOI:** 10.3390/vetsci10020162

**Published:** 2023-02-18

**Authors:** Hanna Lindqvist, Tonje Dominguez, Ragnhild Dragøy, Yunpeng Ding, Lena Burri

**Affiliations:** 1 Department of Animal Environment and Health, Faculty of Veterinary Medicine and Animal Science, Swedish University of Agricultural Sciences, 532 23 Skara, Sweden; 2 Aker BioMarine Antarctic AS, 1366 Lysaker, Norway

**Keywords:** docosahexaenoic acid, dog, eicosapentaenoic acid, krill meal, omega-3 index, premium dog food

## Abstract

**Simple Summary:**

For pets, as for humans, dietary inclusion of long-chain omega-3 fatty acids is recommended for disease prevention and improved health. However, many diets for dogs do not contain sufficient amounts of these fatty acids and fall short of achieving high blood omega-3 levels. This is reflected in the diagnostic health tool called Omega-3 Index (O3I). In this study, O3I levels were measured at baseline in 45 dogs fed a commercial premium diet and compared to O3I levels reached when the dogs were fed with diets containing different omega-3 sources at low inclusion levels, i.e., fish meal/oil, flaxseed cake and krill meal. After four weeks of treatment, the data showed that the highest O3I increase was observed in the 3% krill meal group, accompanied by the lowest arachidonic acid to eicosapentaenoic acid ratio as a measure for immunomodulatory effects. Hence, by using the O3I, this study provides an option for dog owners to measure the impact their pet food has on their dogs’ health and if needed, how to adjust it with the right omega-3 supplement.

**Abstract:**

(1) Background: it is only the longer chain omega-3 polyunsaturated fatty acids (n-3 PUFAs), eicosapentaenoic acid (20:5n-3, EPA), and docosahexaenoic acid (22:6n-3, DHA) and not the shorter chain α-linolenic acid (ALA, 18:3n-3) that have been linked to health benefits. (2) Methods: 45 dogs divided into three groups were first given premium dry food for 38 days (baseline). The O3I was then used as a diagnostic tool to provide a measure of the sum of EPA + DHA in red blood cell membranes given as a percentage of all fatty acids. The dogs were subsequently fed with either krill meal (krill), fishmeal/oil (fish) or flaxseed cake (flax) included in raw food providing daily 416 mg EPA + DHA (971 mg ALA), 513 mg EPA + DHA (1027 mg ALA) and 1465 mg ALA (122 mg EPA + DHA), respectively. (3) Results: the average baseline O3I level of all dogs was low (1.36%), warranting n-3 supplementation. After four weeks, O3I levels were significantly increased in the krill (from 1.36 ± 0.44 to 2.36 ± 0.39%) and fish (from 1.35 ± 0.22 to 1.9 ± 0.35%) groups (*p* < 0.001). No significant modification of the O3I was detected in the flax animals. (4) Conclusions: only marine n-3 PUFAs resulted in a significantly increased O3I, with dietary krill meal providing the highest increase.

## 1. Introduction

Quantities of eicosapentaenoic acid (20:5n-3, EPA) and docosahexaenoic acid (22:6n-3, DHA), the long-chain omega-3 polyunsaturated fatty acids (n-3 PUFAs) that are important for normal growth and disease prevention, as well as for the treatment of cardiovascular, renal, gastrointestinal, orthopedic, dermatological, retinal and immune ailments in dogs [1,2,3,4,5,6], vary considerably in commercial dog foods [7]. While therapeutic diets address higher needs for EPA + DHA and the dosages for dogs range between 50 and 220 mg/kg body weight depending on the condition [8,9], many maintenance diets fail to reach the recommended allowance. As per the recommendation published in the Nutrient Requirements of Dogs by the National Research Council (NRC), growing dogs require 0.13 g/1000 kcal metabolizable energy (ME) and fully-grown dogs 0.11 g/1000 kcal ME of EPA and DHA, respectively [10]. Moreover, the Canine Nutrition Expert Subcommittee of the Association of American Feed Control Officials (AAFCO) determined that 0.05% of EPA + DHA on a dry matter basis (0.1 g/1000 kcal ME) for growing and reproductive dogs is the dietary minimum [11].

Unfortunately, labelling legislation does not require differentiation between shorter and longer chain PUFAs, which can be confusing for consumers [12]. Plant-based oils from flaxseed (flax), canola, walnut and soybean oils contain the shorter chain n-3 fatty acid, α-linolenic acid (ALA, 18:3n-3). ALA provides only indirect health benefits via its conversion to the longer chain n-3 PUFAs [13,14]. However, in mammals, the conversion efficiency is low due to the addition of a double bond by the Δ6-desaturase enzyme, which is the rate-limiting reaction [15]. It is therefore important to include long-chain n-3 PUFAs in the diet. These are commonly found in marine sources such as fatty fish (e.g., menhaden, anchovies, herring and mackerel) crustaceans, and algae. 

The Omega-3 Index (O3I) calculated from a drop of whole blood dried on filter paper provides an easy tool to evaluate the impact of a specific diet on a pet’s EPA + DHA levels as described in a recent study [16]. The study established a conversion factor to translate EPA + DHA measured in whole blood into the O3I for cats and dogs. This factor has previously been well established in humans [17] and is now available for cats and dogs too [16]. The O3I expresses the content of EPA + DHA in red blood cell (RBC) membranes as a proportion of all RBC fatty acids given as a percentage [18]. As such it has a strong correlation with general n-3 status in tissues [19,20]. In humans, it is not only used as a biomarker for long-term n-3 PUFA intake, but also as a potential risk indicator for coronary heart disease [18]. It is expected that also in pets, an increased O3I will positively influence health, however optimal levels have not yet been determined for dogs and await further investigation. A wide range from 0.3 to 7.0%, as observed in random veterinary dog samples, suggests that there is room for improvement in many dogs [16].

The aim of the work was therefore to demonstrate that feeding a premium commercial dog food might not increase the O3I to high levels and compare the efficiency of three different n-3 sources, i.e., from fish, krill and flaxseed to achieve O3I increases. The study presents the O3I as an easy diagnostic tool to provide indications of a dog’s long-term intake of the beneficial n-3 PUFAs, EPA + DHA. It further emphasizes the importance to distinguish between shorter and longer chain n-3 PUFAs and to choose marine- over plant-based supplementation solutions, such as from fish and krill.

## 2. Materials and Methods

### 2.1. Ethics

The study dogs were accustomed to having their blood taken on a regular basis due to their frequent participation in dog sled races and the samples were collected at their own kennel to minimize discomfort. Guidelines by the International Animal Ethics Committee were fully respected, but preceding approval was not required according to the Norwegian regulation of animal experimentations, since the study was a comparison of different feeds, where no adverse effects were expected (described in paragraph 2).

### 2.2. Animals and Diet 

A group of 45 Alaskan Huskies with ages ranging from 2 to 8 years (average of 4.3 years) and a body weight of 22 to 36 kg (average of 27.6 kg), were assigned to one of three dietary treatments after they were sorted for gender and age (Table 1). For sleep, shelter and relaxation, the dogs were bound to individual outdoor, wooden dog houses in proximity to each other to facilitate the ability to see and vocalize. The dogs had ad libitum access to drinking water and were active for one hour per day, usually in a walking machine at the kennel. 

The trial was performed during the low-training period in summer 2021 (starting May 21), thereby representing the dietary needs of active dogs with estimated energy requirements of 2000 kcal ME/day [21].

During the first 38 days of the trial, a premium commercial high energy dry food containing 31% crude protein, 23% crude fat, 36% carbohydrates, 5% ash and 3734 kcal/kg food of ME was given to all dogs. During this period the 45 dogs were fed 400–500 g food daily corresponding to 1494–1867 kcal ME, divided into two meals. This diet was used to represent a widely used commercial food for active dogs involved in sports such as hunting, agility and sled racing and provide a low-O3I-baseline in the dogs. However, since the company producing this food was not open to generate dry food with different n-3 sources, the food was switched to the one of another company specializing in raw food. After 38 days, the dogs were therefore given frozen raw food supplemented with different n-3 sources (Table 2) according to their assigned groups for four weeks (Table 1).

All treatment diets were composed to contain approximately 13% protein, 12% fat and 11% carbohydrates as fed with an energy content of 2000 kcal (8.4 MJ)/kg food (Table 3). When calculated on a dry matter basis, diet 1 (krill) was prepared to contain 3% krill meal supplied by Aker BioMarine Antarctic AS (QRILL^TM^ Pet, Lysaker, Norway) corresponding to 440 mg EPA + DHA per kg food. Diet 2 (fish) was calculated to contain the same amount of EPA + DHA per kg food by including 2% fish meal (Pelagia AS, Bergen, Norway) and 0.4% fish oil (Salmoil^®^, Imazo AB, Vara, Sweden) instead of krill. Diet 3 (flax) contained neither krill nor fish and was thus expected to contain no EPA + DHA. Instead, 1.1% flaxseed cake (Forsbecks AB, Skänninge, Sweden) was added to provide 440 mg extra ALA per kg food compared to the diets, krill and fish. Minor deviations were seen in the analysed diet composition (Table 3).

All dogs in each group consumed on average 1 kg raw food/day divided into two meals, which corresponded to approximately 2000 kcal. The analysed daily intake of EPA + DHA and ALA in the different treatments was 416 mg EPA + DHA (971 mg ALA), 513 mg EPA + DHA (1027 mg ALA) and 1465 mg ALA (122 mg EPA + DHA) in krill, fish and flax treatments, respectively (Table 4).

### 2.3. Sample Collection

A veterinarian examined all the dogs before taking blood samples at baseline and at the end of study. Blood samples were collected venously from the front limb at baseline on July 4 and after 30 days on 4 August 2022. The dogs were weighed at baseline and at the end of the treatment period on August 31. 

### 2.4. Food Analysis

The treatment diets were freeze-dried before analysis and samples were sent to Nofima BioLab, Fyllingsdalen, Norway for analyses of fat, protein, dry matter, ash, fibre and fatty acid profiles as described previously [22]. In short, crude fat was analysed by the method of Bligh and Dyer [23] and fatty acids methyl esters were analysed by gas liquid chromatography with the AOCS Official Method Ce 1b-89 [24]. Nitrogen was analysed using the Kjeldahl method (Kjeltech Auto Analyser, Tecator, Höganäs, Sweden) and crude protein calculated as N × 6.25 [25]. Crude fibre was analysed by a modified version of ISO 5498 based on acid and alkaline hydrolysis. Dry matter was determined by drying at 105 °C until constant weight, and ash levels were obtained after flame combustion and incineration at 550 °C.

### 2.5. Blood Analysis

Gas chromatography with flame ionization detection was used to analyse for fatty acids from dried blood spots to be able to calculate the O3Is as described previously at Omega Quant (Stirling, Scotland) [26].

### 2.6. Statistical Analysis

ANCOVA was used to test the overall effect of diets, by including the baseline O3I values and treatment in the linear model. In addition, in order to detect the difference on each treatment, pairwise comparison was made with Dunnett adjustment. The Wilcoxon matched-pairs signed rank test was used to identify statistical differences in each group from before and after intake of the different diets. Calculations were performed using the statistical program R [27].

## 3. Results and Discussion

A total of 45 dogs (24 males and 21 females) were divided into three groups of 15 dogs. The dogs received either additional EPA + DHA supplementation from fishmeal/oil (fish) or krill meal (krill), or additional ALA was included into their diet from flaxseed cake (flax) to give a calculated daily intake of 1.8 g of n-3 PUFAs (Table 4). The recommended allowance of dietary EPA + DHA was calculated according to the NRC guidelines and was 30 mg/kgBW^0.75^ or 110 mg/1000 kcal ME [10]. The krill and fish diets contained sufficient amounts of EPA + DHA, whereas the flax diet was insufficient in EPA + DHA (Table 5). Food consumption and body weight remained constant over the study period and at the end of study, the average weights were 28.2, 26.9 and 27.8 kg in the krill, fish and flax groups, respectively.

### 3.1. Omega-3 Index 

**Figure 1:**
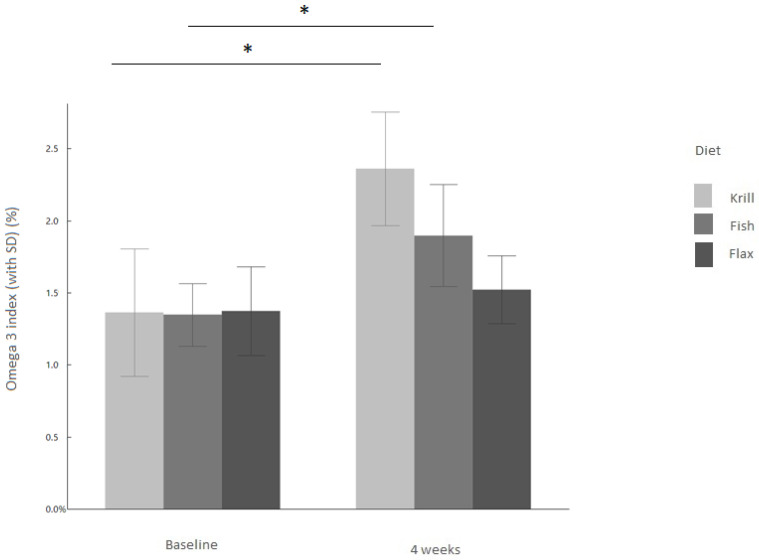
Omega-3 Index with SD (%) for krill, fish and flax groups (*n* = 45). Asterisk indicates a significant difference between baseline and after four weeks of feeding. The comparison test was carried out using the Wilcoxon matched-pairs signed rank test.

After 38 days of feeding with the commercial premium diet, the O3I was not significantly different in the three groups and ranged between 1.35–1.37% (Figure 1 at baseline). These levels were lower than baseline O3I levels in previous published studies, with an average index of 1.7% [28] and 3.9% [29] in non-competing sled dogs, and >5% pre-race in competing sled dogs [30]. In another study, where samples from different dog species from routine care of animals being seen at a veterinary emergency hospital were taken, the OI3 ranged from 0.3 to 7% [16]. In comparison, in human samples, the OI3 ranged between 2 and 12% [16] and a value of 8% or higher is considered optimal to reduce the risk of death from coronary heart disease [18]. 

After 38 days the feeding regime was switched from commercial premium diet to diets containing krill, fish or flax seed diets which were given for four weeks. The O3I increased significantly (*p* < 0.001) in the krill group by 82%, from 1.36 ± 0.44 to 2.36 ± 0.39, and in the fish group by 42.38% from 1.35 ± 0.22 to 1.9 ± 0.35 (*p* < 0.001) (Figure 1). No significant changes in the O3I were detected in the flax-fed animals (baseline: 1.37 ± 0.31; end: 1.52 ± 0.24; *p* = 0.061).

Krill meal made from Antarctic krill (*Euphausia superba*) contains the majority of n-3 PUFAs in the form of phospholipids [31], whereas the PUFAs derived from fish are in triglyceride form. The phospholipid form has been suggested to increase tissue integration of n-3 PUFAs, which has been shown in several species such as dogs [29], piglets [32], baboons [33], mice [34], rats [35] and humans [36]. The results of this study therefore reflect previous observations that favour phospholipid n-3 delivery molecules.

Moreover, this study clearly showed that a diet containing flax cake with shorter chain n-3 does not increase the O3I in dogs, while long-chain marine sources such as krill and fish resulted in significant increases. These results are in agreement with other studies in dogs, where no significant increase of EPA + DHA could be observed after ALA supplementation [13,37].

### 3.2. n-6/n-3 and Arachidonic Acid/EPA Ratios

**Figure 2:**
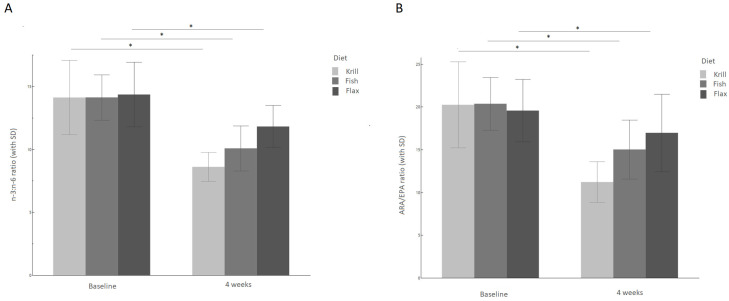
n-6/n-3 and ARA/EPA ratios (with SD). (**A**) n-6 to n-3 ratio (with SD) for the krill, fish and flax groups after four weeks of feeding (*n* = 45). (**B**) ARA/EPA ratio (with SD) for the krill, fish and flax groups after four weeks of feeding (*n* = 45). Asterisk indicates significant difference between baseline and end of study for the treatment group. The comparison test is carried out using the Wilcoxon matched-pairs signed rank test. Abbreviations: ARA: arachidonic acid, EPA: eicosapentaenoic acid.

When EPA + DHA increases in RBC membranes it implies that other fatty acids will decrease accordingly, as will their bioactive derivatives. Indeed, the study found that arachidonic acid (ARA, 20:4n-6), an n-6 PUFA, decreased in RBC membranes with all diets, which contributed to a decreased n-6 to n-3 ratio in following order: krill < fish < flax with ratios declining with 37% (krill), 29% (fish) and 17% (flax) (Figure 2a). This reduction might positively influence the health of dogs, since lower n-6 and higher n-3 levels will shift the balance towards a more anti-inflammatory immune state by reduced production of ARA-derived pro-inflammatory mediators [38].

Likewise, the ratio of ARA/EPA, as a cellular indicator of inflammation [39], decreased most in the krill group with 40%, followed by 26% in the fish and 14% in the flax group (*p* < 0.001) (Figure 2b). 

## 4. Conclusions

The O3I represents n-3 dietary habits over a longer time frame and informs on the effect a dog’s diet has on the body’s EPA + DHA status. It facilitates to distinguish between different n-3 sources and to choose the most effective one. Inasmuch, it became clear that the O3I of dogs after feeding with the tested commercial premium diet was low and most likely insufficient to achieve a lower level of health risk. On the other hand, only a short duration (four weeks) of supplementation of krill and fish was enough to significantly increase the O3I in dogs fed the marine sources of long-chain n-3 PUFAs, which could benefit dogs through reduced inflammation. In addition, the phospholipid form of n-3 PUFAs from the 3% krill diet, led to a 40% higher O3I in the dogs at the end of study, when compared to the fish diet. Moreover, fatty acid ratios that can ultimately impact inflammation and overall health were most improved in the krill group, suggesting that krill meal can be a valuable addition to dog food. 

In contrast, the shorter chain n-3 ALA from flaxseed was not an effective precursor for EPA + DHA. If, however, ALA might have other nutritional benefits besides conversion, such as for the improvement in skin and coat scores [40], was not part of this study and should be addressed in further investigations.

## Figures and Tables

**Table 1:** Group characteristics of the three treatment groups.

	Krill	Fish	Flax
*Gender*			
Males	8	8	8
Females	7	7	7
Age (years)	4 (2–8)	4 (2–8)	4 (2–8)
Weight (kg)	28.5 (22–36)	26.9 (22–33)	27.5 (22.2–34.4)

**Table 2:** Ingredient composition (%) of the three treatment diets.

Ingredient	Krill	Fish	Flax
Beef (meat including lung, tripe and fat)	40.0	42.6	40.9
Chicken necks	12.7	8.1	15.0
Swine trimmings	4.0	3.0	4.0
Maize gluten	3.4	3.6	3.5
Oatmeal	6.7	6.8	5.6
Wheat and barley mix	5.5	5.8	6.0
Rice	2.0	2.0	2.0
Wheat bran	1.0	1.0	1.0
Water from boiling of rice and porridge	22.0	24.0	19.7
Monocalcium phosphate	0.7	0.6	0.7
Calcium carbonate	0.2	0.2	0.3
Potassium chloride	0.2	0.2	0.2
Natrium chloride	0.2	0.2	0.2
Vitamin premix	0.2	0.2	0.2
Choline Chloride	0.1	0.1	0.1
Krill meal	1.2		
Fish oil		0.2	
Fish meal		1.5	
Flaxseed cake			0.5

**Table 3:** Analysed nutritional composition of the three treatment diets as fed.

Analysed Nutrient Composition	Krill	Fish	Flax
Dry matter (%)	37.70	37.10	34.30
Crude protein (%)	13.70	13.90	13.20
Crude fat (%)	13.90	13.20	12.50
*Fatty acids (%)*			
C 14:0	0.39	0.35	0.27
C 16:0	3.27	3.08	2.86
C 16:1	0.35	0.32	0.24
C 18:0	2.11	2.08	2.11
C 18:1	4.83	4.70	4.22
C18:2	0.71	0.76	0.78
C 18:3 (ALA)	0.10	0.10	0.15
C 18:4	0.01	-	-
C 20:5 (EPA)	0.03	0.03	0.01
C 22:5	0.03	0.03	0.02
C 22:6 (DHA)	0.01	0.03	
EPA + DHA (g/kg)	0.42	0.51	0.12
n-3 (g/kg)	1.80	1.80	1.83
n-6 (g/kg)	7.77	8.09	8.30
n-6/n-3	4.05	4.39	4.39
Crude fiber (%)	0.70	0.30	0.60
Crude ash (%)	2.30	2.30	2.30
NFE (%)	7.10	7.40	5.70
Kcal ME */kg	2081	2039	1881

* Metabolizable energy (ME) was calculated with the formula recommended by FEDIAF for foods of vegetable or animal origin in their natural state, kcal ME = (4 × % crude protein) + (9 × % crude fat) + (4 × % NFE) [21].

**Table 4:** Calculated nutritional average daily intake (g) of treatment diets.

Analysed Nutrient Composition	Krill	Fish	Flax
Amount (g)	1000.00	1000.00	1000.00
Water (g)	623.00	629.00	657.00
Crude protein (g)	137.20	139.27	132.14
Crude fat (g)	138.77	131.66	125.11
*Fatty acids (g)*			
C 14:0	3.88	3.46	2.68
C 16:0	32.70	30.80	28.60
C 16:1	3.46	3.20	2.44
C 18:0	21.09	20.80	21.13
C 18:1	48.29	46.98	42.25
C18:2	7.07	7.57	7.81
C 18:3 (ALA)	0.97	1.02	1.46
C 18:4	0.13	-	-
C 20:5 (EPA)	0.28	0.26	0.12
C 22:5	0.28	0.26	0.24
C 22:6 (DHA)	0.14	0.26	-
EPA + DHA	0.42	0.51	0.12
n-3	1.80	1.80	1.83
n-6	7.77	8.10	8.30
n-6/n-3	4.05	4.39	4.39
Crude fiber (g)	7.47	3.04	6.03
Crude ash (g)	22.80	22.83	22.84
NFE (g)	70.76	74.20	56.58
Calcium (g)			
Phosphorous (g)			
Kcal ME	2081	2039	1881

**Table 5:** Recommended daily intake and daily metabolic bodyweight (BW^0.75^) intake of ALA and EPA/DHA in the treatment diets in comparison to the commercial premium diet [10].

	Rec’d Daily Intake	Krill	Fish	Flax	Commercial Premium
*Daily intake*					
ALA (mg)		971	1027	1465	1891
EPA + DHA (mg)		416	513	122	0
*Daily intake/kg BW^0.75^*					
ALA (mg/kg BW^0.75^)	14	79	87	122	157
EPA + DHA (mg/kg BW^0.75^)	30	34	44	10	0
*Daily intake/1000 kcal*					
ALA/ 1000 kcal ME (mg)	110	467	504	779	1085
DHA + EPA/ 1000 kcal ME (mg)	110	200	252	65	0

## Data Availability

The data generated from this study are available upon request to and approval by the authors.

## References

[1] Abba C., Mussa P.P., Vercelli A., Raviri G. (2005). Essential fatty acids supplementation in different-stage atopic dogs fed on a controlled diet. J. Anim. Physiol. Anim. Nutr..

[2] Bauer J.E., Heinemann K.M., Lees G.E., Waldron M.K. (2006). Retinal functions of young dogs are improved and maternal plasma phospholipids are altered with diets containing long-chain n-3 polyunsaturated fatty acids during gestation, lactation, and after weaning. J. Nutr..

[3] Fritsch D., Allen T., Dodd C., Jewell D., Sixby K., Leventhal P., Hahn K. (2010). Dose-titration effects of fish oil in osteoarthritic dogs. J. Vet. Intern. Med..

[4] Mueller R., Fieseler K., Fettman M., Zabel S., Rosychuk R., Ogilvie G., Greenwalt T. (2004). Effect of omega-3 fatty acids on canine atopic dermatitis. J. Small Anim. Pract..

[5] Smith C.E., Freeman L.M., Rush J.E., Cunningham S.M., Biourge V. (2007). Omega-3 fatty acids in Boxer dogs with arrhythmogenic right ventricular cardiomyopathy. J. Vet. Intern. Med..

[6] Trepanier L. (2009). Idiopathic inflammatory bowel disease in cats: Rational treatment selection. J. Feline Med. Surg..

[7] Ahlstrøm Ø., Krogdahl A., Vhile S.G., Skrede A. (2004). Fatty acid composition in commercial dog foods. J. Nutr..

[8] Bauer J.E. (2011). Therapeutic use of fish oils in companion animals. J. Am. Vet. Med. Assoc..

[9] Lenox C., Bauer J. (2013). Potential adverse effects of omega-3 fatty acids in dogs and cats. J. Vet. Intern. Med..

[10] Council N.R. (2006). Nutrient Requirements of Dogs and Cats.

[11] Officials A.o.A.F.C. (2019). Model Bill and Regulations.

[12] Turchini G.M., Nichols P.D., Barrow C., Sinclair A.J. (2012). Jumping on the omega-3 bandwagon: Distinguishing the role of long-chain and short-chain omega-3 fatty acids. Crit. Rev. Food Sci. Nutr..

[13] Bauer J.E., Heinemann K.M., Bigley K.E., Lees G.E., Waldron M.K. (2004). Maternal diet α-linolenic acid during gestation and lactation does not increase docosahexaenoic acid in canine milk. J. Nutr..

[14] Chen H., Deng G., Zhou Q., Chu X., Su M., Wei Y., Li L., Zhang Z. (2020). Effects of eicosapentaenoic acid and docosahexaenoic acid versus α-linolenic acid supplementation on cardiometabolic risk factors: A meta-analysis of randomized controlled trials. Food & function.

[15] Calder P.C. (2013). Omega-3 polyunsaturated fatty acids and inflammatory processes: Nutrition or pharmacology?. Br. J. Clin. Pharmacol..

[16] Harris W.S., Jackson K.H., Carlson H., Hoem N., Dominguez T.E., Burri L. (2022). Derivation of the Omega-3 Index from EPA and DHA Analysis of Dried Blood Spots from Dogs and Cats. Vet. Sci..

[17] von Schacky C. (2011). The Omega-3 Index as a risk factor for cardiovascular diseases. Prostaglandins Other Lipid Mediat..

[18] Harris W.S., Von Schacky C. (2004). The Omega-3 Index: A new risk factor for death from coronary heart disease?. Prev. Med..

[19] Fenton J.I., Gurzell E.A., Davidson E.A., Harris W.S. (2016). Red blood cell PUFAs reflect the phospholipid PUFA composition of major organs. Prostaglandins Leukot. Essent. Fat. Acids.

[20] Metcalf R.G., Cleland L.G., Gibson R.A., Roberts-Thomson K.C., Edwards J.R., Sanders P., Stuklis R., James M.J., Young G.D. (2010). Relation between blood and atrial fatty acids in patients undergoing cardiac bypass surgery. Am. J. Clin. Nutr..

[21] Fediaf (2020). Nutritional Guidelines for Complete and Complementary Pet Food for Cats and Dogs.

[22] Dessen J.-E., Weihe R., Hatlen B., Thomassen M.S., Rørvik K.-A. (2017). Different growth performance, lipid deposition, and nutrient utilization in in-season (S1) Atlantic salmon post-smolt fed isoenergetic diets differing in protein-to-lipid ratio. Aquaculture.

[23] Bligh E.G., Dyer W.J. (1959). A rapid method of total lipid extraction and purification. Can. J. Biochem. Physiol..

[24] Firestone D. (1992). AOCS Official Method Ce-1b-89, Fatty Acid Composition by GLC: Marine Oils, Urbana, IL. https://myaccount.aocs.org/PersonifyEbusiness/Store/Product-Details?productId=111788.

[25] Kjeldahl J. (1883). Neue methode zur bestimmung des stickstoffs in organischen körpern. Z. Für Anal. Chem..

[26] Harris W.S., Polreis J. (2016). Measurement of the omega-3 index in dried blood spots. Ann. Clin. Lab. Res..

[27] Team R.C. R: A Language and Environment for Statistical Computing. R Foundation for Statistical Computing, Vienna, Austria. http://www.R-project.org.

[28] Dominguez T.E., Kaur K., Burri L. (2021). Enhanced omega-3 index after long-versus short-chain omega-3 fatty acid supplementation in dogs. Vet. Med. Sci..

[29] Burri L., Heggen K., Storsve A.B. (2020). Higher omega-3 index after dietary inclusion of omega-3 phospholipids versus omega-3 triglycerides in Alaskan Huskies. Vet. World.

[30] Burri L., Wyse C., Gray S.R., Harris W.S., Lazzerini K. (2018). Effects of dietary supplementation with krill meal on serum pro-inflammatory markers after the Iditarod sled dog race. Res. Vet. Sci..

[31] Burri L., Hoem N., Monakhova Y.B., Diehl B.W. (2016). Fingerprinting krill oil by 31P, 1H and 13C NMR spectroscopies. J. Am. Oil Chem. Soc..

[32] Liu L., Bartke N., Van Daele H., Lawrence P., Qin X., Park H.G., Kothapalli K., Windust A., Bindels J., Wang Z. (2014). Higher efficacy of dietary DHA provided as a phospholipid than as a triglyceride for brain DHA accretion in neonatal piglets. J. Lipid Res..

[33] Wijendran V., Huang M.-C., Diau G.-Y., Boehm G., Nathanielsz P.W., Brenna J.T. (2002). Efficacy of dietary arachidonic acid provided as triglyceride or phospholipid as substrates for brain arachidonic acid accretion in baboon neonates. Pediatr. Res..

[34] Rossmeisl M., Macek Jilkova Z., Kuda O., Jelenik T., Medrikova D., Stankova B., Kristinsson B., Haraldsson G.G., Svensen H., Stoknes I. (2012). Metabolic effects of n-3 PUFA as phospholipids are superior to triglycerides in mice fed a high-fat diet: Possible role of endocannabinoids. PLoS ONE.

[35] Graf B., Duchateau G., Patterson A., Mitchell E., Van Bruggen P., Koek J., Melville S., Verkade H. (2010). Age dependent incorporation of 14C-DHA into rat brain and body tissues after dosing various 14C-DHA-esters. Prostaglandins Leukot. Essent. Fat. Acids.

[36] Ramprasath V.R., Eyal I., Zchut S., Jones P.J. (2013). Enhanced increase of omega-3 index in healthy individuals with response to 4-week n-3 fatty acid supplementation from krill oil versus fish oil. Lipids Health Dis..

[37] Mueller R.S., Fettman M.J., Richardson K., Hansen R.A., Miller A., Magowitz J., Ogilvie G.K. (2005). Plasma and skin concentrations of polyunsaturated fatty acids before and after supplementation with n-3 fatty acids in dogs with atopic dermatitis. Am. J. Vet. Res..

[38] Simopoulos A.P. (2004). Omega-6/omega-3 essential fatty acid ratio and chronic diseases. Food Rev. Int..

[39] Magalhães R., Guardiola F.A., Guerreiro I., Fontinha F., Moutinho S., Serra C.R., Olsen R.E., Peres H., Oliva-Teles A. (2022). Immunomodulatory effect of different dietary ARA/EPA/DHA ratios in gilthead sea bream (Sparus aurata) juveniles after infection with Photobacterium damselae subsp. piscicida. Aquac. Res..

[40] Bauer J.E. (2007). Responses of dogs to dietary omega-3 fatty acids. J. Am. Vet. Med. Assoc..

